# Styrene maleic acid encapsulated raloxifene micelles for management of inflammatory bowel disease

**DOI:** 10.1186/s40169-017-0157-2

**Published:** 2017-08-03

**Authors:** Khaled Greish, Safa Taha, Anfal Jasim, Sara Abd Elghany, Ameera Sultan, Ali AlKhateeb, Manal Othman, Fang Jun, Sebastien Taurin, Moiz Bakhiet

**Affiliations:** 1 Department of Molecular Medicine, College of Medicine and Medical Sciences, Princess Al-Jawhara Centre for Molecular Medicine, Arabian Gulf University, Manama, Kingdom of Bahrain; 2 Department of Anatomy, College of Medicine and Medical Sciences, Arabian Gulf University, Manama, Kingdom of Bahrain; 3 Department of Histology, Faculty of Medicine, Assiut University, Asyut, Egypt; 4 Department of Pharmacology and Oncology, Sojo University, Kumamoto, Japan; 5 Department of Obstetrics and Gynecology, University of Utah, Salt Lake City, USA

**Keywords:** Styrene maleic acid (SMA), Raloxifene, Inflammatory bowel disease (IBD), Nanomedicine, Enhanced permeability and retention effect (EPR), Inflammatory cytokines

## Abstract

**Background:**

Inflammatory bowel disease (IBD) comprises a group of disorders that manifest through chronic inflammation of the colon and small intestine. Although the exact cause of IBD is still unclear, dysfunctional immunoregulation involving overproduction of inflammatory cytokines such as TNF-α, and IL-6 have been implicated in pathogenesis. Current therapy relies on immunosuppression, cytotoxic drugs, and monoclonal antibodies against TNF-α. These classes of drugs have severe side-effects, especially when used for long duration. Our previous work with raloxifene, a selective estrogen receptor modulator, has shown that the drug, and to a greater extent its micellar formulation, has a significant suppressive effect on NF-κB, an essential immune-regulator. This finding directed the current work towards testing the anti-inflammatory and immunomodulatory effects of raloxifene using cell lines, as well as testing the potential use of the styrene maleic acid (SMA) micelles loaded with raloxifene (SMA-Ral) against dextran sulfate sodium (DSS) induced colitis in an in vivo model of IBD.

**Results:**

Treatment of MCF-7 cells with TNF-α was shown to protect the cells from the cytotoxic effect of raloxifene (42 vs. 10% cell death, with TNF-α. Treating CaCo-2 cells with both free and SMA-Ral improved cell survival after exposure to 2% DDS with significantly higher protection with SMA-Ral. Treatment of U-937 with SMA-Ral and free-Ral resulted in down-regulation of TNF-α, IL-1β, IL-6, and MIP1α, with greater inhibition of the SMA-Ral, compared to free Ral. Balb/c mice treated with raloxifene and SMA-Ral showed weight gain at 14 days, compared to the control group (122, and 115% respectively). Treatment with raloxifene prevented DSS-induced diarrhea in 6/6 of free raloxifene treated mice and in 5/6 mice treated with SMA-Ral. Control group of DSS-treated mice showed average colon length of 7.4 cm compared to 13 cm in the control group. The average colon length was 12.3 and 11.5 cm for raloxifene and SMA-Ral treated groups, respectively. Furthermore, inflammatory cytokines such as IL-6 and TNF-α were reduced in serum of animals treated with free-Ral and SMA-Ral.

**Conclusions:**

Raloxifene and its micellar formulation warrants further studies to understand their effect on the treatment of colitis.

**Graphical abstract:**
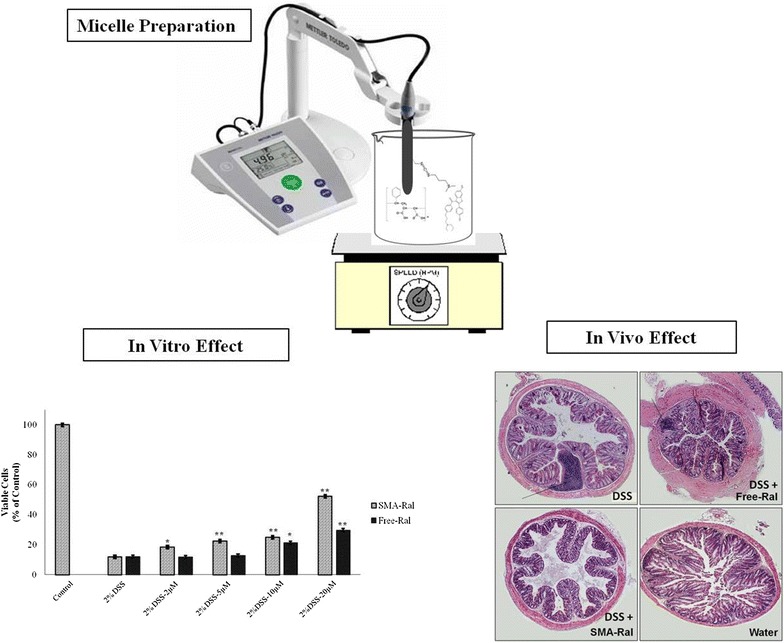
SMA-Raloxifene preparation and its in vivo and in vitro effect on colitis

## Background

Inflammatory bowel disease (IBD) is an elusive disease that lacks a clear understanding of the pathogenesis as well as an established curative therapy. The IBD term encompasses both ulcerative colitis (UC) and Crohn’s disease (CD). Both conditions share the presence of alternating relapsing inflammation and remissions, with different clinical and pathophysiological indexes [1].

IBD usually starts at young adulthood and leads a course of remissions and relapses for the remaining lifetime of affected patients. The disease prevalence is rising worldwide and seems to affect developed countries more than developing countries. In North America, 1.5 million patients are diagnosed with IBD, and 2.2 million are having the disease in Europe [2–5].

IBD had been classified as cell mediate type IV hypersensitivity reaction to unspecified microbial agents with specific genetic predisposition involving nucleotide-binding oligomerization domain containing 2 (NOD-2) polymorphisms [6].

Common to both UC and CD is the over expression of inflammatory mediators, specifically TNF-α, IL- 6 and NFκB, and this is the main rationale for using corticosteroids, cytotoxic drugs and the biological response modifiers [7–9]. However, the efficacy of these agents for the treatment of IBD remains poor and is associated with significant side effects. Estrogens have also been suggested to play a role in the development of IBD pathogenesis. Estrogen receptors α and β are expressed along the intestinal track as well as by immune cells [10]. Furthermore, estrogens have been demonstrated to have proinflammatory effects in DSS mediated colitis [11].

Raloxifene is a second generation selective estrogen receptor modulator (SERM). Raloxifene was approved for the reduction of the risk of invasive breast cancer in postmenopausal women and management of postmenopausal women with osteoporosis [12].

We have previously demonstrated that raloxifene can exert anticancer activity against different breast and prostate cancer cell lines independently of its SERM activity. One pathway that has been significantly inhibited involved the expression of NFκB [13–15].

NFκB is a protein complex that controls the expression of IL-1β, TNF, IL-6, IL-8, ICAM-1 and co-stimulatory molecules, including CD40, CD80, CD86 and the inducible T-cell co-stimulator ICOS. All of these pro-inflammatory hallmark molecules have been implicated in IBD. Thus, we hypothesized that inhibition of NFκB by raloxifene could have a role in the management of IBD [16]. However, raloxifene effect is limited in vivo by low bioavailability (2%) due to poor solubility, extensive metabolism, and being prone to efflux mechanisms of various transporters such as multidrug resistance-related proteins, or organic anion transporter. Therefore, we formulated raloxifene nanomicelles using poly (styrene-co-maleic acid) (SMA) as a nanodelivery platform to improve water solubility, protect the drug from metabolism, and efflux mechanisms and potentially improve its anti-inflammatory effect against IBD models [17–19].

## Materials and methods

### Materials

Raloxifene hydrochloride (99% purity), cumene terminated poly(styrene-co-maleic anhydride) with an average Mn∼1600, N-(3-dimethylaminopropyl)-N-ethylcarbodiimide hydrochloride (EDAC), sulforhodamine B, dextran sulfate sodium salt (DSS were obtained from Sigma-Aldrich Ltd (Germany). Quantikine ELISA kits including Mouse IL-6 Immunoassay, Cat. No. M6000B, and Mouse TNF-α Immunoassay, Cat. No. MTA00B were purchased from R&D systems) (USA).

### Methods

#### Synthesis of SMA-Ral micelles

Synthesis of SMA-Ral micelles was done as described previously [13]. In brief; SMA was hydrolyzed in 1 M NaOH at 70 °C. Once soluble, SMA solution was adjusted to pH 5. Next, raloxifene-HCl was solubilized in dimethyl sulfoxide (DMSO) and added gradually to the SMA solution along with the distilled water-solubilized EDAC. The prepared solution left on constant stirring for 20 min at pH 5. Afterward, the solution was adjusted to pH 11 with 0.1 M NaOH with continued stirring to allow the formation of SMA micelles and encapsulation of raloxifene. After 30 min, the pH was readjusted to 7.4 and the micelle suspension was ultrafiltered four times using a Labscale™ ultrafiltration system mounted with a Pellicon^®^ XL filter 10 kDa (Merck Millipore, Auckland, New Zealand). The SMA-Ral powder was then obtained by freezing the concentrated micelle solution overnight at −80 °C followed by lyophilization.

#### Characterization of SMA-Ral micelles

Raloxifene content of the prepared SMA micelles was determined as described before [13] and expressed as wt% of Ral in the final micelle compared to the total weight of recovered SMA-Ral. Both size and polydispersity index (PDI) of the SMA-Ral micelles were measured using the Malvern ZEN3600 Zetasizer Nano series (Malvern Instruments Inc., Westborough, MA) following the aforementioned procedures in [13]. Raloxifene release from SMA micelles was assessed by dialysis system [13] and the release % was determined as follow:

$${\text{\% release}} = \frac{{{\text{amount outside the bag }}({\text{at defined time}})}}{{{\text{amount inside the bag at t}} = 0\, {\text{min}}}} \times 100$$

### Cell culture

Human U937, MCF-7 and CaCo2 cell lines were obtained from American Type Culture Collection (ATCC, USA). Cells were grown at 37 °C in 5% CO_2_ and 100% humidified atmosphere in Advanced RPMI 1640 (1×) growth medium (gibco^®^ by life technologies™, USA) supplemented with 10% (v/v) fetal bovine serum (FBS) (SIGMA life sciences, USA), penicillin-streptomycin antibiotics (SIGMA life sciences, USA) and l-glutamine 200 mM (100×) (gibco^®^ by life technologies™, USA). Cells passage was performed every 3–4 days and suspensions of each cell line were produced from confluent cultures using 10× TrypLE™ (gibco^®^ by life technologiesTM, USA).

### Effect of SMA-Ral on inflammatory cytokines

Equal quantities of U937 cells (1 × 10^5^ cell/well) were seeded into 24-well tissue culture plates (Corning, New York). After 24 h, cells were treated with different concentrations of SMA-Raloxifene (2, 5, 10, and 20 µM), free Raloxifene and control. Plates were then incubated at two different intervals, 24 and 72 h. Cell pellets/of treated and untreated cells were suspended in 350 µl of buffer RLT + β-mercaptoethanol (10 µl β-ME/1 ml of buffer RLT). Afterward, RNA samples from the different cells were extracted following the protocol of QIAamo^®^ RNA Blood Mini Kit from QIAGEN. Next, 1 µg of each RNA was reverse-transcribed into cDNA samples by using high-capacity cDNA reverse transcription kit (Applied Biosystems, Lithuania) in a thermal cycler (Perkin Elmer Amp Gene 9700, USA) programmed to perform 3 steps as follow: 25 °C for 10 min, 37 °C for 120 min, 85 °C for 5 s. Quantification and purification of all cDNA samples were assessed by using NanoDrops spectrophotometer (Thermo Scientific). Genes of interest (IL-6, TNF-α, IL_10, MCP-1, MIP-1α, IL-1β and βactin) were then amplified from the resulted cDNA samples by performing PCR using specific primers and thermal cycler (Perkin Elmer Amp Gene 9700, USA) programmed to perform an initial denaturation step of 95 °C for 10 min followed by 40 cycles of denaturation at 95 °C for 15 s, 60 °C for 30 s, a final extension of 60 °C at 30 s and a subsequent hold at 4 °C. Finally, resulted amplicons (10 µl) were visualized by 1.5% agarose gel.

### Detection of TNF-α and Ral interactions

Equal quantities of MCF-7 cells (1 × 10^5^ cell/well) were seeded directly into 96-well tissue culture plates (Corning, New York, USA). After 24 h, cells were treated with different concentrations of TNF-α (10 pg, 100 pg/ml), raloxifene (10 μM) and a combination of both. After, the plate was incubated for 72 h at 37 °C in 5% CO_2_ and later fixed with trichloroacetic acid (TCA) overnight at 4 °C. Sulforhodamine B (SRB) assay was performed to determine cytotoxicity of the different treatment conditions [20]. OD of the solubilized dye in each well was determined at 450 nm with 570 nm as a reference wavelength. OD mean ± SD were calculated and expressed as percentage of cell viability when compared to controls.

### Effect of SMA-Ral on DSS induced toxicity

A total count of CaCo-2 (1 × 10^5^ cells/well) was cultured in 96-well tissue culture plate (Corning, New York, USA). Cells were all treated with 2% DSS plus different concentrations of free raloxifene (2, 5, 10, 20 µM) or SMA- Ral (2, 5, 10, 20 µM), with one line of non-treated cells as a control. After, the plate was incubated for 72 h at 37 °C in 5% CO_2_ and fixed with (TCA) overnight. SRB assay was performed to determine cell viability [20]. OD of the cells-bound dye at each well was determined at 450 nm with 570 as a reference wavelength using a. OD mean ± SD were calculated and cell viability percentages were then determined and compared according to the OD results.

### In vivo effect

#### Effect of SMA-Ral in DSS-induced colitis in mice

Female Balb/c mice (age: 5–6 weeks) were separated into four groups (n = 5 per group) and given 3% DSS in drinking water for 14 days. Control mice group received water without DSS. At day 7, two groups were orally treated either by free Ral or SMA Ral for 3 days with a dose equivalent to 5 mg/kg. Mice weight and clinical conditions (stool consistency) were monitored daily and at the end of the treatments weight change was expressed as a ratio of day 14 weights to day 0 weights of the same mouse and compared to the control group. After 2 weeks, mice were sacrificed and colons length and weight were measured and compared. Colon tissue samples were fixed in 10% formalin and undergo histological scoring by H&E staining to detect and compare cellular infiltration, depletion and damage between the different mouse groups.

#### Mice IL-6 and TNF-α immunoassay

Blood samples from all treated and control mouse groups were collected and centrifuged to obtain plasma samples. Plasma samples were used to quantitatively determine levels of IL-6 and TNF-α cytokines by the sandwich enzyme immunoassay technique using Quantikine ELISA kits and following the recommendation of the manufacturer. The enzyme reaction yields a blue product that turned yellow when the stop solution is added. The intensity of the color was measured to determine the optical density in each well—using a microplate reader set at 450 nm, wavelength correction at 570 nm. Samples values were then read off the standard curve that created by four parameter logistic (4-PL) curve-fit.

## Results

### SMA-Ral micelles synthesis and characterization

SMA-Ral was successfully synthesized and characterized as indicated in [13]. The micelles water solubility was 10.6 mg/ml and the weight ratio of raloxifene over SMA indicated a drug loading percentage of 20%. Size detection showed micelles of 65.34 ± 30.89 nm mean diameter with a polydispersity index of 0.135. Charge of the micelles was near neutral with a zeta potential of −0.0165 mV. On the other hand, after 36 h, release rate measurements at physiological pH, intestinal pH and gastric fluid pH was 32, 25 and 73%, respectively.

### In vitro effect of SMA-Ral

#### Effect of SMA-Ral on inflammatory cytokines

**Fig. 1:**
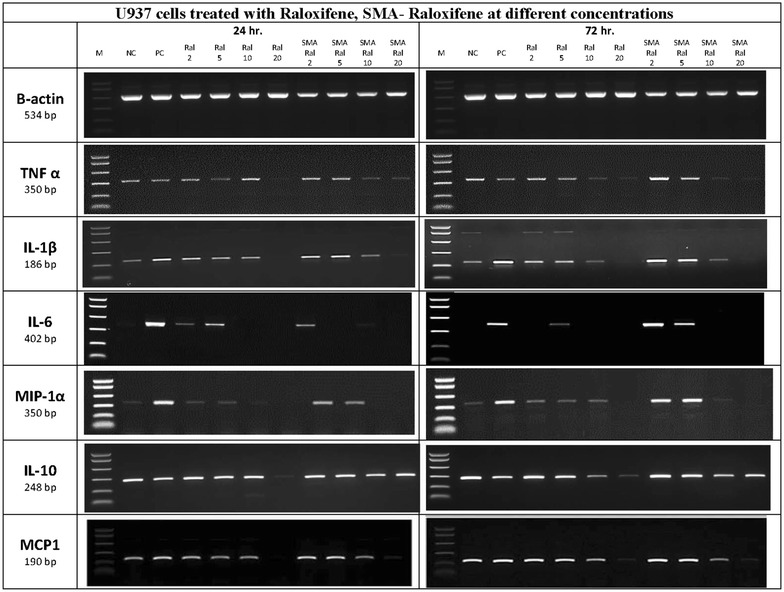
cDNA samples (10 µl) of different inflammatory cytokines expressed by U937 cells, treated with different concentration of free or SMA Ral and visualized on 1.5% agarose gel

In order to detect the effect of SMA-Ral on the expression of different inflammatory cytokines, U937 cells were treated with different concentrations of SMA-Ral and free raloxifene. After 72 h, both SMA-Ral and the free raloxifene resulted in comparable down regulation of TNF-α, IL-1β and MCP1 when given at concentrations of 10 or 20 μM (Fig. 1). The two formulations have also totally suppressed the expression of both MIP-1α and IL-6 cytokines at 20 µM. However, SMA-Ral has no effect on IL-10 when compared to free raloxifene which slightly inhibited IL-10 expression after 72 h at 20 μM.

### Detection of TNF-α and Ral interactions

**Fig. 2:**
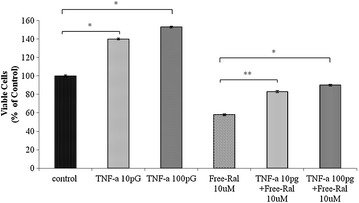
Average MCF7 cell number following 72 h of TNF-α, free-Ral and combination of both. All data are expressed as mean ± SD. * denotes p ≤ 0.05 and ** denotes p ≤ 0.001 as determined by t test

In vitro treatment of MCF7 cells with TNF-α (10 pg, 100 pg) has resulted in around 40% increase in cells proliferation with a little more effect of the 100 pg TNF-α concentration (Fig. 2). However, while free Ral (10 µM) treatment resulted in about 42% cell death in MCF-7 cells when compared to the control conditions. A combination of both Ral and TNF-α (10 or 100 pg) was shown to protect from the cells from the cytotoxicity mediated by of raloxifene (only 17 and 10% cell death, respectively) proposing an antagonist effect of TNF-α against the cytotoxic effect of the free Ral (Fig. 2).

### Effect of SMA-Ral on DSS induced toxicity

**Fig. 3:**
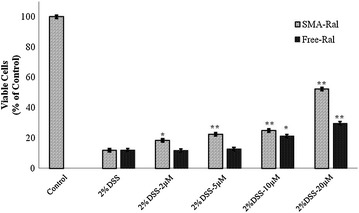
Average CaCo2 cell number following 72 h of free and SMA Ral treatments. All data are expressed as mean ± SD. * denotes p ≤ 0.05 and ** denotes p ≤ 0.001 as determined by t test and in relative to the 2% DSS treated cells

Treatment of CaCo-2 cells with 2% DSS has reduced cell viability by 90% after 72 h incubation. Although simultaneous treatment of the Caco2 cells with 2% DSS and 20 µM of free raloxifene or SMA-Ral for 72 h did not prevent cytotoxicity, it led to 60 and 80% increment in cell survival respectively (Fig. 3).

### In vivo effect

#### Effect of SMA-Ral in DSS-induced colitis in mice

**Fig. 4:**
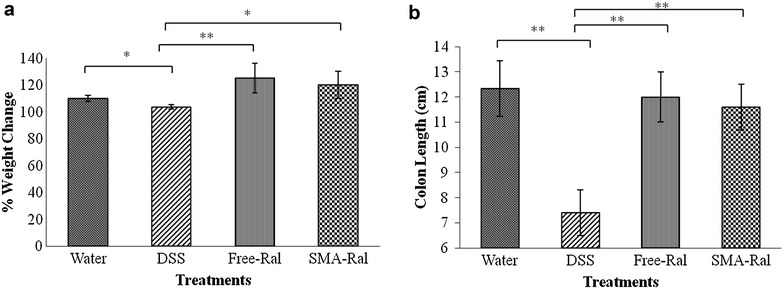
**a** Effect of free and SMA Ral treatments compared to DSS on mouse weight. The average weight change for each cage was calculated relative to the first day. **b** Results of colon length measurements in the differently treated mouse groups. All data are expressed as mean ± SD. * denotes p ≤ 0.05 and ** denotes p ≤ 0.001 as determined by t test

**Fig. 5:**
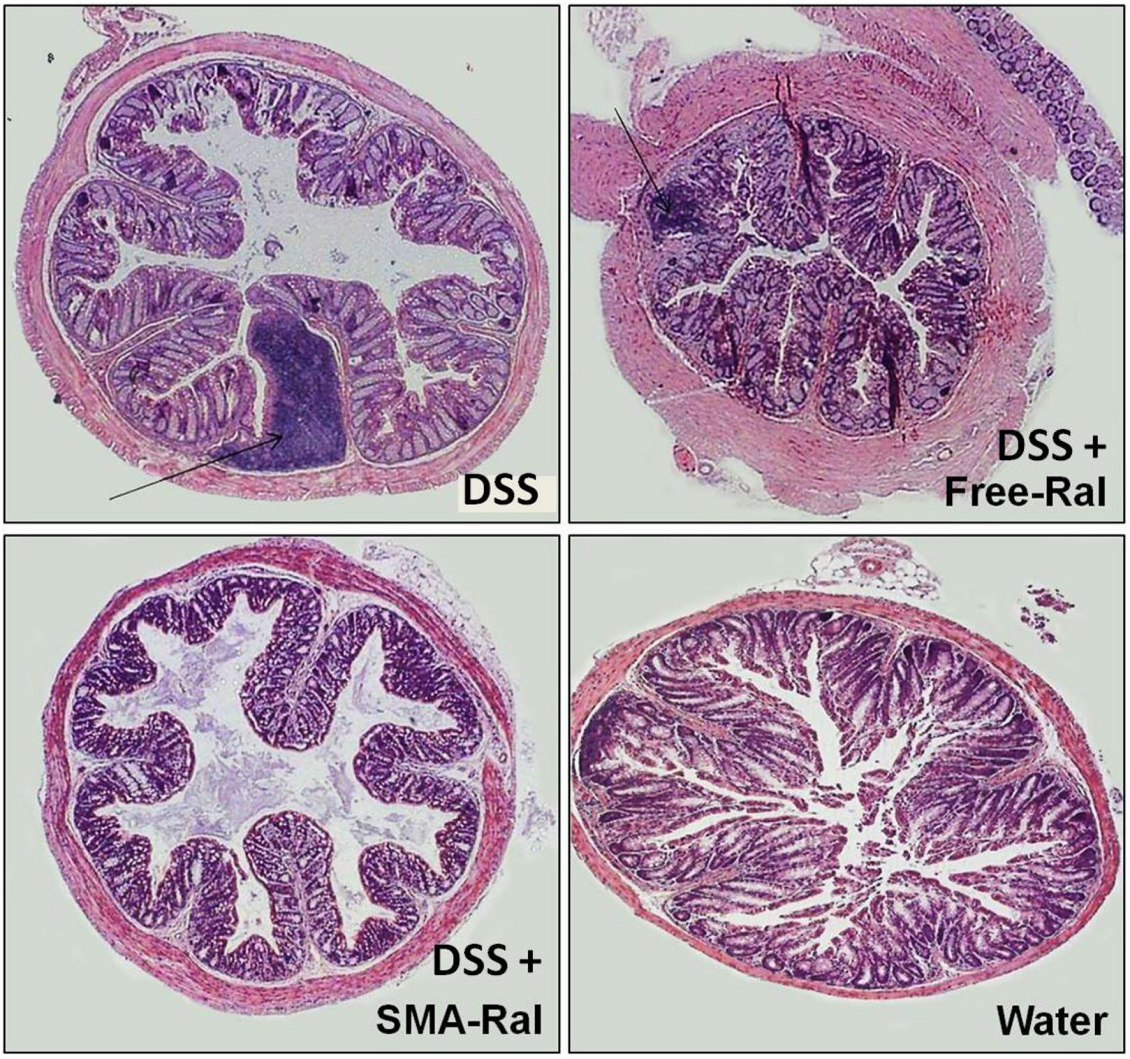
Distal colon sections (×10, *scale bar* 500 µm) showing the histological features of the differently treated mice groups. *Arrows* point to inflammatory cells

To evaluate the anti-inflammatory effect of orally administered SMA-Ral micelles, female Balb/c mice were orally treated by free Ral or SMA Ral after colitis induction by DSS. Compared to the control mice group, weight gain was clearly observed after 14 days in the two groups treated with free Ral or SMA-Ral (122, and 115% respectively) (Fig. 4a). Treatment with free Ral prevented the DSS-induced diarrhea in 5/5 mice and in 4/5 mice treated with SMA-Ral (Appendix). Also, control group of DSS-treated mice showed average colon length of 7.4 cm compared to 13 cm of the control group treated with water (Fig. 4b). However, for the other two groups, sequential treatment with 3% DSS and free-Ral or SMA-Ral administration showed a protective advantage of the two raloxifene formulations against colon atrophy in which average colon length was 12.3 and 11.5 cm in free-Ral- and SMA-Ral treated mice, respectively. In addition, histological scoring of the collected colon tissue exposed significant differences between the different mice groups (Fig. 5). DSS treated mouse samples indicated highly inflamed colon tissue with extensive mononuclear cells infiltrations in comparison to the tissue obtained from unexposed mice. Colon tissues obtained from both free and SMA Ral treated groups clearly demonstrate colon recovery after treatment.

#### Mice IL-6 and TNF-α immunoassay

**Fig. 6:**
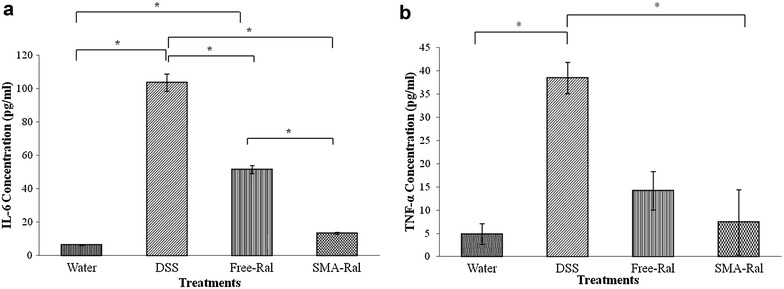
**a** Effect of free and SMA Ral treatments compared to DSS or water treatment on mouse IL6 expression levels. **b** Effect of free and SMA Ral treatments compared to DSS or water treatment on mice TNF-α expression levels. All data are expressed as mean ± SD. * denotes p ≤ 0.05 and ** denotes p ≤ 0.001 as determined by t test

The analysis of blood samples collected from the DSS-treated mice showed an increase of IL-6 level of 194% in colitis-induced mice compared to unexposed mice (Fig. 6a). However, mice treated with free Ral or SMA-Ral has shown lower IL-6 levels (88 and 53%, respectively) compared to the only DSS treated mice. Furthermore, mice group treated with 2% DSS had 87.5% TNF-α level increase when compared to unexposed mice (Fig. 6b). TNF-α level decreased by nearly two-fold and three-fold when colitis-induced mice were treated with free Ral or SMA-Ral, respectively. For both IL-6 and TNF-α, SMA-Ral more efficiency reduced the levels of both cytokines to levels near the normal range.

## Discussion

The incidence and prevalence of IBD have been increasing worldwide across pediatric and adult populations [5]. Recent epidemiological data from United States documented a positive correlation between the use of oral contraceptive pills (OCPs) and IBD. Compared to non-users of OCPs, current users of OCPs have a 2.82 fold higher risk to develop CD. Even though the mechanistic relationship has not been yet elucidated, this finding clearly suggests a role for SERM in modifying the course of IBD [21]. Our previous experience with Raloxifene has documented its ability to reduce the expression of NF-κB making a plausible role for SERMs in management of IBD.

In this work, we compared the use of free raloxifene and the SMA-Ral on the reduction of inflammation in IBD model in vitro and in vivo. SMA-Ral was used to improve the low bioavailability of raloxifene. The encapsulation of raloxifene protects the drug from the direct activity of metabolizing enzymes and efflux system. Furthermore the size of SMA-Ral (65.34 ± 30.89 nm) could concentrate in inflammatory tissues due to the enhanced permeability and retention (EPR) effect. The EPR effect is known for both tumor and inflammatory tissues where selective and pronounced extravasation of large molecules can ensue due to contraction of endothelial cells in blood vessel of inflammatory tissues, resulting in the formation of large gaps that allows the extravasation of macromolecules larger than 7 nm [22].

Furthermore, micellar encapsulation could provide and sustained release form. SMA-Ral showed a release rate of 25% at intestinal pH (value) after 36 h, this in addition to the ability of SMA micelles system to cross the intestinal epithelium, would be advantageous in improving the dosage frequency of Ral [23]. As shown in Fig. 1, U937 cells treated with free-Ral and SMA-Ral, showed significant reduction of the level of TNF-α, IL-1β, MCP1, MIP-1α, and IL-6 but no effect on IL-10 level. U937 cells were chosen to recapitulate the behavior of differentiated monocytes that infiltrate actively inflamed intestinal tissues. The results clearly show that the raloxifene as a free or micellar formulation reduces the expression of inflammatory mediators to a similar extent.

In order to test whether raloxifene antagonized TNF-α, we treated MCF-7 breast cancer cells with TNF-α at 10, and 100 pg. TNF-α treatment promoted a higher cell proliferation at these two doses as expected. TNF-α is a double edge sword in tumor development as it can promote cancer cell progression, or induce apoptosis depending on cellular interaction and the quantity of the cytokine. TNF-α activates signaling pathways involving NF-κB and c-Jun N-terminal kinase (JNK). NF-κB is a major cell survival signal that is anti-apoptotic while sustained JNK activation contributes to cell death [24]. It has been longely established that NF-κB pathway regulates proinflammatory cytokine production, leukocyte recruitment and cell survival [25]. In this study, raloxifene significantly improved cell survival of cells treated with TNF-α, (Fig. 3). Whether this effect is a mere additive effect of both molecules or a true antagonizing effect of TNF-α by inhibiting NF-κB remains to be investigated. We further tested if raloxifene could alter cell survival of CaCo2 cells that simulate intestinal cells, we challenged the cells with 2% DSS and then added ascending doses of Ral and SMA-Ral (2–20 µM), as shown in Fig. 3 both free Ral and SMA-Ral significantly improved the cell survival after DSS treatment.

We attribute this effect to the anti-inflammatory effect of Ral that could be mediated through reduction of IL-1β, TNF-α and IL-8, all can be released by inflamed colonic cells [26].

The efficacy of free-Ral and SMA-Ral for the protection against intestinal mucosal inflammation was tested in vivo. DSS model of induced colitis was used because of its rapid effect, simplicity, reproducibility and controllability [27]. DSS is negatively charged due to its sulfate groups, the drug can interact with medium-chain-length fatty acids in the colon and could erode negatively charged colonic mucosal cells resulting in enhancing the colonic cell permeability to colonic bacteria, hence the DSS shows its peak inflammatory effect on the colon, which bears the highest bacterial content in the GI tract [28]. In administering the DSS at 3% in Balb/C mice, there was not much weight loss in control groups, however, both free and SMA-Ral treated mice showed significant weight gain (Fig. 4a). Weight gain is known to develop early after DSS treatment; however this usually is reversed as inflammation progressed. SMA-Ral and free-Ral could possibly simulate this early stage of inflammation resulting in persistent weight gain at 14 days [27].

However, the most direct evidence of the effect of Ral and its formulation can be acquired by measuring colon length and by histological examination after H&E staining. As shown in Fig. 4b, colon length was significantly shorter in DSS group (Appendix), while both SMA and free Ral could reverse this effect. Furthermore, as shown in Fig. 5, treatment with Ral and to a greater extent SMA-Ral could inhibit the massive cellular infiltration and destruction of the colonic mucosal cells. Direct evidence of the resulting effect could be acquired by measuring both IL-6 and TNF-α in the serum of different treatment groups. As shown in Fig. 6 SMA-Ral had the highest effect in reducing both inflammatory mediators, and this reduction was statistically significant in case of IL-6 level.

TNF-α is a key molecule central to IBD pathogenesis. Accumulated research has demonstrated that TNF-α activation can result in epithelia hyper-permeability, neoangiogenesis, augmentation of inflammatory cytokine production by macrophages and T cells. TNF-α can results in breaching the intestinal barrier and produce massive tissue destruction through inducing myofibroblasts to produce matrix metalloproteinases (MMPs). Further it can results in T cell resistance to apoptosis which eventually drive the inflammatory process to chronic phase through a process involving activation of NF-κB [29].

IL-6 production is produced both macrophages and CD4^+^ T cells in patients with IBD. Upon binding of its receptors, IL-6–sIL-6R activate antigen presenting cells and T cells. In addition the IL-6–sIL-6R complex reduces apoptosis of mucosal T cells and activates pro-inflammatory cytokine production by these cells. Experimental blockade of IL-6 signaling with monoclonal antibodies was effective in suppressing chronic intestinal inflammation in mouse models, which suggests IL-6 as a potential therapeutic target in management of IBD [30, 31].

Indeed, the introduction of anti-TNF-α monoclonal antibodies to the clinical milieu of IBD therapy resulted in marked clinical and histological improvement in treated patients. However, TNF-α monoclonal antibodies could be associated with low incidence of severe adverse effects such as induction of malignant lymphomas, infections, congestive heart failure, a lupus-like syndrome, induction of auto-antibodies, injection site reactions, and high incidence of tuberculosis [32].

Considering the results all together, SMA-Ral was marginally more effective in managing DDS induced colitis compared to free Ral. However, considering the biodistribution of the encapsulated micelle and the free drug, this can be advantageous in favor of the micellar Ral. The free drug would be able to exert the SERM effect on various body tissues. The SMA formulation on the other hand have a restricted biodistribution profile. As shown in many of our previous publications, SMA micelles reaching the blood stream will be uptaken mostly by the reticuloendothelial cells in the spleen and liver [19]. In this respect, the micelle would be potentially safer than the free drug. This is especially true in case of male patients and premenopausal females.

Our results clearly indicate the efficacy of Ral and to a higher extent SMA-Ral in reducing both the culprit cytokines in tested DSS induced colitis model. The wide suppression of multiple inflammatory mediators through single molecule could be highly advantageous in IBD management. On the other hand, apart from in the risk for venous thrombo-embolism, the chronic and wide use of Ral was not associated with other side effects. The lower incidence of the side effects as well the reduce cost, could be advantageous for pursuing further clinical trials on the effect of Ral on IBD.

## Conclusions

We successfully formulated and characterized SMA-Ral micelles. Both free drug and micellar formulation showed attention of multiple inflammatory signaling pathways in vitro, and could protect CaCo2 colon cell line against DSS induced death. In vivo testing of the drugs significantly resulted in inhibition of both IL-6 and TNF-α level in treated mice. The micellar formulation showed narrow but significant improvement in reduction of IL-6, while its higher suppression rate of TNF-α was not statistically significant. The current results warrant the clinical exploration of Ral and SMA-Ral in management of IBD.

## Figures and Tables

**Fig. 7:**
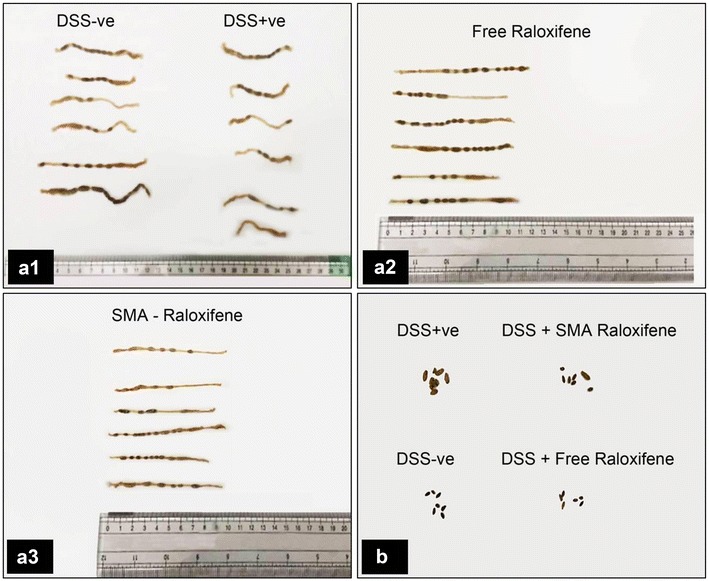
**a1**–**a3** Effect of free and SMA Ral treatments compared to DSS on colon length. **b** Effect of free and SMA Ral treatments compared to DSS on stool consistency

## Appendix

See Fig. 7.
